# Ethanol-Extracted Cameroonian Propolis Counteracts Tamoxifen-Induced Endometrial Hyperplasia by Modulating Apoptosis and Proliferation-Regulating Proteins in the Ovaries of Intact Wistar Rats

**DOI:** 10.1155/2022/2684742

**Published:** 2022-04-13

**Authors:** Charline Florence Awounfack, Stéphane Zingué, Bruno Koumabas, Alain Brice Tueche, Charlotte Mungho Tata, Fernand-Nestor Tchuenguem Fohouo, Dieudonné Njamen, Derek Tantoh Ndinteh

**Affiliations:** ^1^Department of Animal Biology and Physiology, Faculty of Science, University of Yaoundé I, P.O. Box 812, Yaoundé, Cameroon; ^2^Department of Medical and Biomedical Engineering, Higher Technical Teachers' Training College, University of Yaoundé I, P.O. Box 886, Ebolowa, Cameroon; ^3^Department of Life and Earth Sciences, Higher Teachers' Training College, University of Maroua, P.O. Box 55, Maroua, Cameroon; ^4^Centre for Natural Product Research, Department of Chemical Sciences, University of Johannesburg, P.O. Box 17011, Doornfontein, Johannesburg 2028, South Africa; ^5^Department of Biological Sciences, Faculty of Science, University of Ngaoundere, P.O. Box 454, Ngaoundere, Cameroon

## Abstract

Tamoxifen is the most common adjuvant that has been widely used in the treatment of positive estrogen receptor (ER+) breast cancer for over 20 years. However, long term exposure to tamoxifen doubles the risk of endometrial cancer. The association of tamoxifen with antiproliferative substances could abrogate its side effects on the endometrium. Recently, we demonstrated that ethanol-extracted Cameroonian propolis (EECP) has chemopreventive effects on ER+ breast cancer in rats. This study evaluated the capability of EECP to counteract tamoxifen-induced endometrial hyperplasia, without altering its effect on the breast. Thirty-six rats of ∼2 months were coadministered either EECP (16.5, 50, and 150 mg/kg BW) or fulvestrant (300 *μ*g/kg BW) and tamoxifen (10 mg/kg BW) for 8 weeks. Afterward, the relative weights and histomorphometry of the uterus, vagina, ovaries, and mammary gland were assessed. The expression of some proteins of proliferation (PCNA), angiogenesis (VEGF), and apoptosis (Bax, Bcl-2, and caspase-3) was measured by immunohistochemistry. Rats that received only tamoxifen had endometrial hyperplasia compared to normal rats. EECP and fulvestrant protected the rats against tamoxifen-induced endometrial hyperplasia. A significant decrease in uterine wet weight (*p* < 0.01); endometrial height (*p* < 0.001); and expression of PCNA, Bcl-2, and VEGF proteins as well as a significant increase in the expression of Bax and caspase-3 proteins was observed in the EECP group compared to the Tamox group. EECP did not change the effects of tamoxifen on the breast. In summary, Cameroonian propolis which is efficacious in preventing breast cancer can also be a good complementary medicine to prevent tamoxifen-induced endometrial cancer in tamoxifen users.

## 1. Introduction

Cancer is a group of diseases involving uncontrolled proliferation and abnormal cell growth with the potential to invade or spread to other parts of the body [1]. It is the second leading cause of mortality worldwide after heart diseases with 19.3 million new cases and 10 million deaths in 2020 [2]. In 2020, breast cancer was the first leading cause of cancer-related deaths in women (626,679 deaths), and it accounts for 30% of all new cancer cases diagnosed in women worldwide (about 2.1 million new cases) [1, 2]. In Cameroon, it is also the most common cancer in women with more than 2000 new cases each year [3]. Several studies have demonstrated that failure in responding to apoptosis signals, severe proliferation, and angiogenesis are essential for the growth, invasion, and metastasis of primary tumors, including breast cancer [4]. To face this threat, many treatments have been developed; they include but are not limited to chemotherapy, radiotherapy, targeted therapy, surgery, and hormonal therapy.

The most common hormonal treatment against ER+ breast cancer in both women and men is tamoxifen [5]. This selective estrogen receptor modulator (SERM) competes with endogenous estrogen for binding ERs in the breast and triggers apoptosis in tumorous cells. It is recognized nowadays that tamoxifen increases the overall survival of patients treated for breast cancer. It could play an important role in chemoprevention of breast cancer in women with high risk factors, but this still remains controversial [6]. Although the benefit of tamoxifen in the management of ER+ breast cancer has been established (low risk of recurrence and contralateral breast cancer), its risk of inducing endometrial cancer has also been recognized [7], thus raising the issue of how to counteract the tamoxifen-induced endometrial hyperplasia. Numerous authors have demonstrated that the chemopreventive effects of tamoxifen could be improved by combining it with substances that could potentiate its antitumor activity or mitigate its side effects on the endometrium [5, 8]. In view of this, many natural substances have been screened to improve the benefit/risk balance of using tamoxifen.

Propolis, so-called “bee glue,” is a strongly adhesive natural resinous mixture collected (from buds, leaves, and bark of plants and pollen) and produced by bees (mainly *Apis mellifera* L.) [9]. Edaphoclimatic factors as well as plant origin can play a key role in the formation of propolis, mainly their molecular composition which greatly depends on the surrounding ecosystem in which the bees nest [10, 11]. This variation in the chemical composition of propolis justifies the constant interest of researchers in propolis from unexplored regions [12, 13]. Previous studies have reported that Cameroonian propolis has antioxidant [14], antibacterial [15], and estrogenic properties [16]. In addition to these properties, the ethanolic extract of Cameroonian propolis, just like propolis from other parts of the world, has recently been shown to have chemopreventive effects against breast cancer induced in rats in comparison to tamoxifen [17–20]. It seemed interesting to us to look at its possible protective effect on the endometrium in patients taking tamoxifen.

## 2. Materials and Methods

### 2.1. Chemicals

The citrate tamoxifen (Mylan^®^) was obtained from MYLAN SAS (Saint-Priest, France). The fulvestrant (ICI 182, 780) was provided by Tocris Bioscience (Bristol, UK). The antibodies anti-Bcl-2 (B cell lymphoma 2), anti-Bax (B cell lymphoma 2 associated X protein), anti-PCNA (proliferating cell nuclear antigen), anti-caspase-3, and anti-VEGF (vascular epidermal growth factor) were obtained from Dickinson Biosciences (Heidelberg, Germany). The anesthetics diazepam (Valium^®^ 10 mg/2 mL) and ketamine (ketamine hydrochloride 50 mg/mL) were obtained from Roche (Fontenay-sous-Bois, France) and Rotexmedica (Trittau, Germany), respectively.

### 2.2. Preparation of Ethanol-Extracted Cameroonian Propolis

The propolis sample used in this study was harvested from modern hives of *Apis mellifera* in Meiganga (Adamawa region, Cameroon) in December 2019 by Professor Nestor-Fernand Tchuenguem Fohouo (University of Ngaoundere, Cameroon) and kindly provided to us.

The ethanolic extract of Cameroonian propolis (EECP) was prepared as described in our previous work which was in accordance with other works [14, 16, 17, 20]. Briefly, 120 g of propolis was dried in a shade, ground, and soaked in 600 mL hydroethanol (70 : 30) for 24 h at ambient temperature. The supernatant was filtered using Whatman paper N°4, centrifuged (at 600 × g for 10 min), and lyophilized using a freeze drier (Christ Beta 1–8 K, Bioblock Scientific, Germany) for 72 h. Finally, a brown powder crude extract of 21 g was obtained and kept dry at 4°C until use. The UPLC-HRMS analysis performed on this same sample showed the presence of caffeic acid derivatives and triterpenoids in EEP, as reported by Zingue et al. [16].

### 2.3. Determination of Doses

The EECP was administered at three doses (16.5, 50, and 150 mg/kg BW) per os with reference to a previous study in our laboratory by Zingue et al. [20]. Indeed, this study, which was aimed at evaluating the chemopreventive effects of EECP, revealed that it prevented the incidence of breast tumors in rats in a dose-dependent manner with an optimal effect at 150 mg/kg BW. Tamoxifen was administered at 10 mg/kg BW per os, a dose reported as effective in inducing endometrial hyperplasia in rats [8]. The pure antiestrogen fulvestrant (ICI 182,780) was administered at a dose of 300 *μ*g/kg BW s.c. as previously described [16].

### 2.4. Animals

Healthy juvenile female Wistar rats aged ∼2 months and weighing ∼150 g were purchased from the breeding facility of the Laboratory of Animal Physiology (University of Yaoundé I, Cameroon). Animals were housed in clean plastic cages at room temperature (∼25°C) under natural illumination (approx. 12 h light/dark). Their access to food and water was ad libitum, with standard soy-free rat chow [corn (36.7%), bone flour (14.5%), wheat (36.6%), fish flour (4.8%), crushed palm kernel (7.3%), sodium chloride (0.3%), and vitamin complex (Olivitazol^®^ - 0.01%)] to eliminate exposure to exogenous estrogen.

Animal housing, care, and experiments were carried out in accordance with directives of the Institutional Ethics Committee of the Cameroon Ministry of Scientific Research and Innovation, which adopted the directives established by the European Union on the care of animals (EEC Council 86/609).

### 2.5. Study Design

After one week of acclimatization, thirty-six (36) rats were randomly allocated to 6 groups (*n* = 6) as follows: Group I (NOR) serving as normal control and Group II serving as negative control (Tamox) both received the vehicle (distilled water); Group III serving as a positive control was treated with fulvestrant at a dose of 300 *μ*g/kg BW s.c. The three remaining groups (IV, V, and VI) were treated with the ethanol-extracted Cameroonian propolis at doses of 16.5, 50, and 150 mg/kg BW. Endometrial hyperplasia was induced by subchronic (56 days) administration of tamoxifen (10 mg/kg) per os to all animals except the NOR control, which received distilled water instead. The animals were weighed weekly from the first day of acclimatization until the end of the experiment. The treatments lasted for 8 weeks; at the end, the animals (∼4 months of age) were submitted to a nonhydric fast for 12 hours and sacrificed under light anesthesia with ketamine and diazepam (10 and 50 mg/kg BW, i.p.). Arteriovenous blood was collected into dry tubes by cervical decapitation and centrifuged at 600 g for 15 min. Estrogen target organs (uterus, vagina, ovaries, and mammary glands) and some organs of interest to the toxicity study (liver, lungs, spleen, kidneys, stomach, and adrenal glands) were removed, trimmed of fat, weighed, and immediately fixed in a formaldehyde-buffered solution.

### 2.6. Histological Analysis

Histological analyses of the uterus, vagina, ovaries, and mammary glands were performed on 5 *μ*m sections of paraffin embedded tissues following hematoxylin–eosin staining. The changes in the histoarchitecture and measurement of the epithelial heights were assessed on photomicrographs obtained by using a light Axioskop 40 microscope equipped with a digital Celestron-44421 camera connected to a computer where the image was transferred and analyzed with ImageJ software.

### 2.7. Immunohistochemical (IHC) Analyses

The same blocks previously prepared for histological analysis were cut to obtain ten (10) sections of the uterus for each protein to be assessed. After deparaffinization and rehydration of sections, a rapid recovery and activation of the antigens were performed by the enzymatic method for 5 min. After two washes with phosphate-buffered saline (PBS), the sections were incubated with 0.3% hydrogen peroxide in methanol for 10 min to neutralize endogenous peroxidase activity. The sections were then washed in PBS, and nonspecific background was reduced by incubating them with goat serum (Sigma Aldrich, Frankfurt, Germany) for 1 h. Afterward, the sections were incubated for 1 h in the dark with the following antibodies: (i) rat anti-PCNA (1 : 5000), (ii) rat anti-Bax (1 : 500), (iii) rabbit anti-Bcl-2 (1 : 200), (iv) rat anti-VEGF (1 : 500), and (v) rabbit-anti-caspase-3 (1 : 200). For the negative control, the primary antibody was replaced by PBS. After 3 consecutive washes in distilled water and 3 others in PBS, the uterine sections were incubated with secondary rabbit antibody for 30 min. After washing with distilled water and PBS, Dako LSAB^®^2 System-HRP (Dako North America Inc., Carpinteria, USA) was applied for immunodetection according to the manufacturer's instructions. The visualization was carried out using a Dakopatts substrate (Glostrup, Denmark) based on diaminobenzidine liquid tetrahydrochloride (DAB) at concentrations suggested by the manufacturer. The sections were counterstained with hematoxylin and mounted in a Canada resin.

The percentage of immunopositive cells was estimated as the ratio of the number of points striking the immunopositive endometrial cells to the number of points impartially striking space reference (all types of endometrial cells). For this, four to five sections of the uterus were analyzed per animal at 400× magnification. Quantitative analyses of tested protein immunopurification were performed using ImageJ software.

### 2.8. Statistical Analysis

Results are presented as means ± standard error of the mean (SEM). One-way analysis of variance (ANOVA) followed by Dunnett's post hoc test for multiple comparisons was used for statistical analysis of data using SigmaPlot software version 11.00. A *p* value less than 0.05 was considered to be statistically significant.

## 3. Results

### 3.1. Effects of EECP on Body Weight

**Figure 1:**
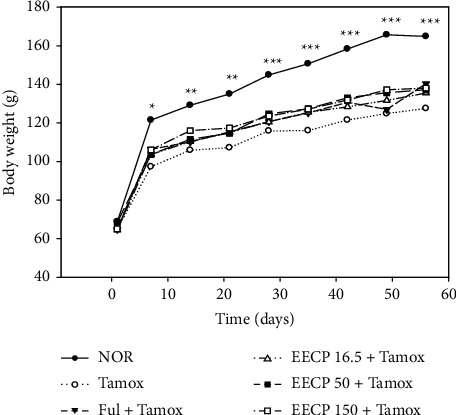
Effects of EECP on body weight of animals after 56 days of treatment. NOR: rats treated with distilled water as normal control; Tamox: rats treated with tamoxifen only at the dose of 10 mg/kg BW as negative control; Ful: rats treated with the pure antiestrogen fulvestrant at the dose of 300 *μ*g/kg BW as positive control; EECP: rats treated with the ethanol-extracted propolis from Cameroon at doses of 16.5, 50, and 150 mg/kg BW as test groups. All rats apart from those of the normal group (NOR) were concomitantly administered tamoxifen at the dose of 10 mg/kg BW for 8 weeks to induce endometrial hyperplasia. Significance compared to Tamox group: ^*∗*^*p* < 0.05, ^*∗∗*^*p* < 0.01, ^*∗∗∗*^*p* < 0.001.

Results depicted in Figure 1 show that all animals were growing normally during the 2 months of experiment. No significant change was observed in body weights of all animals receiving tamoxifen; however, rats in the normal group were bigger than all rats treated with tamoxifen. In fact, there was a gradual increase in body weights of rats in the normal group which became significant from day 8 (*p* < 0.05) to day 56 (*p* < 0.001) compared to animals receiving tamoxifen.

### 3.2. Effects of EECP on the Uterus

#### 3.2.1. Effects of EECP on Uterine Wet Weight

**Figure 2:**
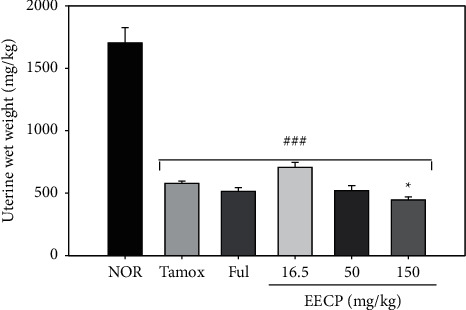
Effects of EECP on uterine wet weight after 56 days of treatment. NOR: rats treated with distilled water as normal control; Tamox: rats treated with tamoxifen only at the dose of 10 mg/kg BW as negative control; Ful: rats treated with the pure antiestrogen fulvestrant at the dose of 300 *μ*g/kg BW as positive control; EECP: rats treated with the ethanol-extracted propolis from Cameroon at doses of 16.5, 50, and 150 mg/kg BW as test groups. All rats apart from those of the normal group (NOR) were concomitantly administered tamoxifen at the dose of 10 mg/kg BW for 8 weeks to induce endometrial hyperplasia. Significance compared to NOR group: ^###^*p* < 0.001; significance compared to Tamox group: ^*∗*^*p* < 0.05.

Figure 2 shows a drastic decrease (*p* < 0.001) of uterine wet weight of all rats treated with tamoxifen as compared to animals in the normal group: 1704.22 ± 121.13 mg/kg for rats in the normal group compared to 577.82 ± 18.84 mg/kg in the negative control group (Tamox). No significant change was noted in the uterine wet weight of different animals treated with tamoxifen, except for those treated with EECP at 150 mg/kg, which had a significant (*p* < 0.01) decrease in uterine wet weight compared to rats of the Tamox group.

#### 3.2.2. Effects of EECP on the Endometrium

**Figure 3:**
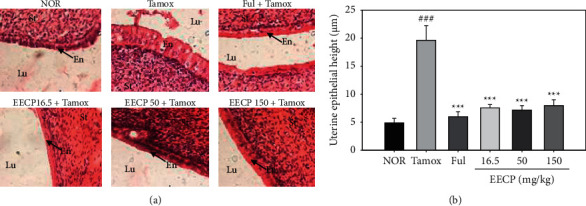
Effects of EECP on photomicrographs (H&E 400×) of the uterus (a) and endometrial height (b) of rats after 56 days of treatment. NOR: rats treated with distilled water as normal control; Tamox: rats treated with tamoxifen only at the dose of 10 mg/kg BW as negative control; Ful: rats treated with the pure antiestrogen fulvestrant at the dose of 300 *μ*g/kg BW as positive control; EECP: rats treated with the ethanol-extracted propolis from Cameroon at doses of 16.5, 50, and 150 mg/kg BW as test groups. All rats apart from those of the normal group (NOR) were concomitantly administered tamoxifen at the dose of 10 mg/kg BW for 8 weeks to induce endometrial hyperplasia. Significance compared to NOR group: ^###^*p* < 0.001; significance compared to Tamox group: ^*∗∗∗*^*p* < 0.001. Lu: lumen of uterus; En: endometrium; St: stroma.

Tamoxifen treatment induced a significant (*p* < 0.001) increase in endometrial size (from 4.91 ± 0.79 *μ*m in normal group to 19.62 ± 2.63 *μ*m in Tamox group) (Figure 3). As expected, the antiestrogen fulvestrant (300 *μ*g/kg) significantly (*p* < 0.001) prevented the tamoxifen-induced endometrial hyperplasia. Interestingly, EECP at all the tested doses, just like fulvestrant, significantly (*p* < 0.001) counteracted the tamoxifen-induced endometrial hyperplasia.

#### 3.2.3. Effects of EECP on the Expression of Some Proteins in the Uterus

**Figure 4:**
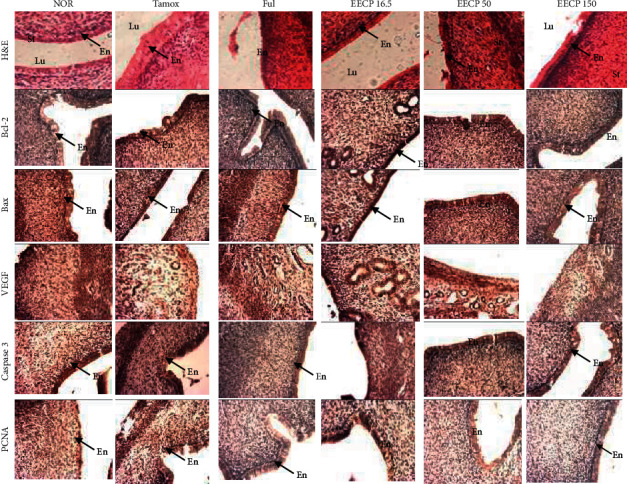
Photomicrographs (H&E 400× and immunohistochemical staining) of the endometrium after 56 days of treatment with EECP. NOR: rats treated with distilled water as normal control; Tamox: rats treated with tamoxifen only at the dose of 10 mg/kg BW as negative control; Ful: rats treated with the pure antiestrogen fulvestrant at the dose of 300 *μ*g/kg BW as positive control; EECP: rats treated with the ethanol-extracted propolis from Cameroon at doses of 16.5, 50, and 150 mg/kg BW as test groups. All rats apart from those of the normal group (NOR) were concomitantly administered tamoxifen at the dose of 10 mg/kg BW for 8 weeks to induce endometrial hyperplasia. Lu: uterine lumen; En: endometrium; St: stroma.

The expression of some proteins of cell proliferation (PCNA), apoptosis (Bax, Bcl-2, and caspase-3), and angiogenesis (VEGF) was analyzed by ICH to determine the effects of EECP on cell proliferation and apoptosis in the endometrium (Figure 4 and Table 1). Photomicrographs of the endometrium of animals treated with tamoxifen showed an overexpression of Bcl-2 and PCNA proteins which was evident in immunohistological sections by a strong dye affinity for chromogen (brown color) compared to the normal group (Figure 4). On the other hand, an underexpression of Bax and caspase-3 proteins was observed in the endometrium of animals treated with tamoxifen compared to normal animals.

The quantitative analyses of protein immunopurification showed a significant increase (*p* < 0.05) in the expression of Bcl-2, VEGF, and PCNA proteins in the endometrium of animals treated with tamoxifen compared to normal animals (Table 1). Moreover, a significant decrease in the expression of the Bax protein was observed in tamoxifen-treated animals. Fulvestrant induced an underexpression of Bcl-2 (not significant) and PCNA (*p* < 0.05) proteins and an overexpression of the proapoptotic Bax protein. EECP at the dose of 16.5 mg/kg did not alter the expression of the tested proteins in the animal endometrium. However, it induced an underexpression of Bcl-2, VEGF, and PCNA proteins at the doses of 50 and 150 mg/kg. The underexpression of the 3 proteins was statistically significant at 150 mg/kg, whereas only the underexpression of VEGF protein was significant at 50 mg/kg. In addition, an overexpression of Bax (significant only at the dose of 150 mg/kg, *p* < 0.05) and caspase-3 [significant at doses of 50 mg/kg (*p* < 0.05) and 150 mg/kg (*p* < 0.01)] proteins was observed following EECP treatment as compared to the animals of Tamox group.

### 3.3. Effects of EECP on the Vaginal Epithelial Heights

**Figure 5:**
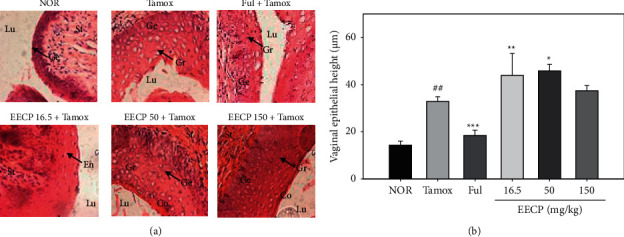
Photomicrographs (H&E staining, 400×) (a) and graphic representation (b) showing the effects of EECP on the vaginal epithelial height. NOR: rats treated with distilled water as normal control; Tamox: rats treated with tamoxifen only at the dose of 10 mg/kg BW as negative control; Ful: rats treated with the pure antiestrogen fulvestrant at the dose of 300 *μ*g/kg BW as positive control; EECP: rats treated with the ethanol-extracted propolis from Cameroon at doses of 16.5, 50, and 150 mg/kg BW as test groups. All rats apart from those of the normal group (NOR) were concomitantly administered tamoxifen at the dose of 10 mg/kg BW for 8 weeks to induce endometrial hyperplasia. Significance compared to NOR group: ^##^*p* < 0.001; significance compared to Tamox group: ^*∗*^*p* < 0.05, ^*∗∗*^*p* < 0.01, ^*∗∗∗*^*p* < 0.001. Co: stratum corneum layer; Lu: vaginal lumen; Gr: stratum granulosa; Ge: stratum germinativum; St: stroma.

Figure 5 shows the photomicrographs of the vagina. Treatment with tamoxifen induced a significant increase (*p* < 0.01) in the vaginal epithelial height as compared to the normal animals (NOR). The antiestrogen fulvestrant significantly abrogated the increase in vaginal epithelial height induced by tamoxifen. On the other hand, EECP caused an increase in vaginal epithelial height at doses of 16.5 (*p* < 0.01) and 50 (*p* < 0.05) mg/kg as compared to animals treated with tamoxifen only.

### 3.4. Effects of EECP on the Mammary Glands

**Figure 6:**
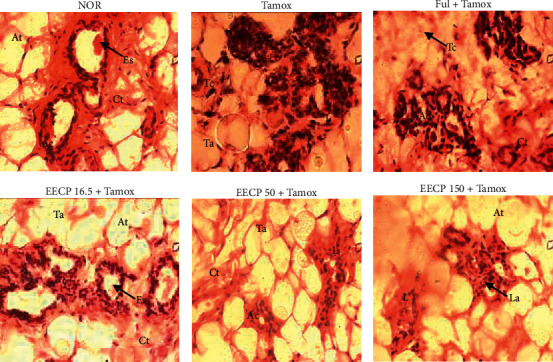
Photomicrographs (H&E staining, 400×) showing the effects EECP on the mammary glands. NOR: rats treated with distilled water as normal control; Tamox: rats treated with tamoxifen only at the dose of 10 mg/kg BW as negative control; Ful: rats treated with the pure antiestrogen fulvestrant at the dose of 300 *μ*g/kg BW as positive control; EECP: rats treated with the ethanol-extracted propolis from Cameroon at doses of 16.5, 50, and 150 mg/kg BW as test groups. All rats apart from those of the normal group (NOR) were concomitantly administered tamoxifen at the dose of 10 mg/kg BW for 8 weeks to induce endometrial hyperplasia. La: lumen of acini; Ac: acini epithelial cell; Es: eosinophil secretion; At: adipose tissue; Ct: conjunctive tissue.

Figure 6 shows that normal rats had normal acini diameter with individualized lumen and normal epithelial cells filled by eosinophil secretions. Animals treated with tamoxifen or tamoxifen + fulvestrant presented a reduction in the size and lumen of acini compared to those of normal animals. Interestingly, the breast lobules and acini of animals treated with EECP at all tested doses were not different from those of tamoxifen-treated rats.

### 3.5. Effects of EECP on the Ovaries

**Figure 7:**
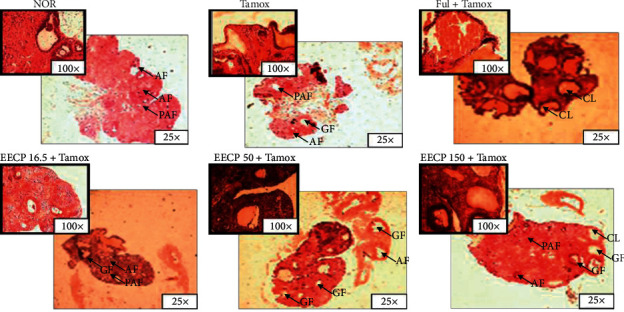
Photomicrographs (H&E staining 400×) of the ovaries of animals treated for 8 weeks with different substances. NOR: rats treated with distilled water as normal control; Tamox: rats treated with tamoxifen only at the dose of 10 mg/kg BW as negative control; Ful: rats treated with the pure antiestrogen fulvestrant at the dose of 300 *μ*g/kg BW as positive control; EECP: rats treated with the ethanol-extracted propolis from Cameroon at doses of 16.5, 50, and 150 mg/kg BW as test groups. All rats apart from those of the normal group (NOR) were concomitantly administered tamoxifen at the dose of 10 mg/kg BW for 8 weeks to induce endometrial hyperplasia. P: primary follicle, PF: primordial follicle, PAF = pre-antral follicle, AF: antral follicle, GF: de Graaf follicle, CL: corpora lutea.

Figure 7 and Table 2 depict the effects of EECP on the ovaries. Rats treated with tamoxifen exhibited a significant (*p* < 0.001) accumulation of primary (from 4 ± 1.63 in the normal group to 16 ± 0 in the Tamox group) and secondary (from 0.93 ± 0.58 in the normal group to 6 ± 1.14 in the Tamox group) follicles compared to normal animals. Moreover, the number of corpora lutea present in the ovary stroma significantly decreased (*p* < 0.01) with tamoxifen treatment. The antiestrogen fulvestrant significantly reduced the number of primary follicles (from 16 ± 0 in the Tamox group to 3 ± 0.81 in rats treated with fulvestrant) and increased (*p* < 0.01) the corpora lutea (from 1.75 ± 0.42 in the Tamox group to 4.74 ± 1.33 in the rats treated with fulvestrant). The EECP significantly (*p* < 0.01) reduced the number of primary follicles as compared to animals treated with tamoxifen only. Moreover, only the dose of 150 mg/kg was able to induce a significant (*p* < 0.01) increase in the number of corpora lutea (from 1.75 ± 0.42 in the Tamox group to 5.2 ± 1.01 in EECP 150).

Two (2) rats out of 6 (33.3%) presented with ovarian cysts in the Tamox group. All rats treated with EECP had ovarian cysts: 1/6 rats at the dose of 150 mg/kg; 2/6 rats at doses of 16.5 and 50 mg/kg.

### 3.6. Effects of EECP on Some Organ Wet Weights

No significant changes were observed in the relative weights of the different organs (Table 3), except for the brain wet weight, which significantly (*p* < 0.05) increased in the Tamox group as compared to the normal group (NOR).

## 4. Discussion

In Cameroon, breast cancer is the most common cancer in women with more than 2000 new cases each year [3]. To combat the disease, many treatments are available nowadays including hormonal therapy directed against ER+ breast cancer [5]. One of the hormonal therapies that is mostly recommended for the management of premenopausal ER+ breast cancer patients is tamoxifen [5]. Although tamoxifen reduces the risk of recurrence and contralateral breast cancer, its long term use is associated with increased incidence of endometrial cancer [7]. Thus, efforts are being made to find substances that could be with tamoxifen to counteract its side effects on the endometrium. In this study, we evaluated the ability of EECP, which is endowed with antiestrogenic and anti-mammary tumor properties in rats [16, 20], to counteract tamoxifen-induced endometrial hyperplasia without altering its effects on mammary glands.

The drastic decrease (*p* < 0.001) in uterine wet weight observed in all rats that received tamoxifen compared to normal rats corroborates the observations of Nuttall et al. [21], who showed that idoxifene [a selective estrogen receptor modulator (SERM)] and tamoxifen are capable of causing significant and dose-dependent decrease in uterine wet weight in rats with an optimal effect at the dose of 15 mg/kg. This observation was also reported for other SERM such as GW5638 and raloxifene [22]. The significant decrease (*p* < 0.001) in uterine wet weight observed in rats cotreated with tamoxifen and EECP at the dose of 150 mg/kg suggests that EECP counteracted the effect of tamoxifen. This is in accordance with the findings of Zingue et al. [16], who reported the *in vitro* antiestrogenic effects of EECP on human embryonic kidney cells (HEK293T) stably transfected with ER*α* and ER*β*. The significant increase (*p* < 0.001) in the endometrial height (hyperplasia) noted in rats that received 10 mg/kg tamoxifen is in agreement with the observations made by numerous authors [5, 7, 8, 23]. Tamoxifen is well known to induce endometrial hyperplasia in rats through genomic (Er*α*) and nongenomic (insulin-like growth factor 1) pathways [7, 24]. According to Feng et al. [24], tamoxifen was also found to promote endometrial hyperplasia by activating protein kinase C delta (PRKCD), which in turn triggers the Nrf2-dependent sequestosome 1 (SQSTM1) transcription. An inverse effect has been observed when coadministering tamoxifen and fulvestrant or EECP. Branham et al. [25] reported similar effects when cotreating Sprague Dawley rats with the tamoxifen and the pure antiestrogen fulvestrant. These results suggest that EECP protects rats against the tamoxifen-induced endometrial hyperplasia probably by competing with tamoxifen for binding to ERs, since EECP has been shown to transactivate ERs [16].

PCNA is a 36-kDa nuclear protein which is known to be expressed during cell cycle and whose synthesis rate is directly correlated with cell proliferation [26]. VEGF is a powerful specific mitogen of endothelial cells which is associated with angiogenesis and tumor invasion [27]. Apoptosis is a fundamental form of physiological cell death which involves two main pathways. The intrinsic mitochondrial pathway is regulated by a balance between proapoptotic (e.g., Bax, Bak, Bad) and antiapoptotic (e.g., Bcl-2 and Bcl-XL) proteins all belonging to the Bcl-2 family [28]. The antiapoptotic proteins block the release of cytochrome C while the proapoptotic proteins induce it. The latter activate a complex called apoptosome, which in turn activates caspase-9 and then caspases-3, 6, and 7 resulting in apoptosis [28]. In this study, apoptosis was evaluated by measuring the expression of proapoptotic (Bax and caspase-3) and antiapoptotic (Bcl-2) proteins. The significant increase (*p* < 0.05) in the expression of PCNA, VEGF, and Bcl-2 proteins and the decrease (*p* < 0.05) in the expression of the Bax protein observed in endometrium of tamoxifen-treated rats as compared to normal rats suggest the onset of endometrial hyperplasia. This observation is in line with the increase in endometrial height. A significant (*p* < 0.05) increase in PCNA and VEGF expression was also reported by Karlsson et al. [29] and Golderg et al. [30], respectively, in tamoxifen-induced endometrial hyperplasia condition. The significant underexpression of PCNA (*p* < 0.05), Bcl-2 (*p* < 0.05), and VEGF (*p* < 0.05 at 50 mg/kg and *p* < 0.01 at 150 mg/kg) proteins and the significant overexpression of Bax (*p* < 0.05) and caspase-3 (*p* < 0.05 at 50 mg/kg and*p* < 0.01 at 150 mg/kg) proteins in the endometrium following EECP treatment just like the pure antiestrogen fulvestrant are in agreement with the results of endometrial height, thus suggesting that EECP acted by inhibiting cell proliferation and triggering apoptosis. In fact, these results are in line with the protective effects of propolis and its polyphenolic compounds as reported by numerous works [17–19].

The cornification of vaginal epithelium is one of the hallmarks of estrogenic activities of tested substances [31]. The significant (*p* < 0.01) increase in vaginal epithelial height observed in rats treated with tamoxifen only resulted in a stratification of the vaginal epithelium revealing three layers (stratum germinativum, stratum granulosum, and stratum corneum), suggesting the onset of the estrus. Several studies have reported the same effect with tamoxifen: antiestrogenic on the mammary glands and estrogenic on the uterus and vagina [32, 33]. The significant increase in vagina epithelial height following cotreatment with tamoxifen and EECP at 16.5 and 50 mg/kg suggests that propolis potentiated the effects of tamoxifen. Similar results have been observed on the vagina of the ovariectomized rats after 3 days of treatment with EECP at doses of 50 and 150 mg/kg [16], confirming the status of phytoestrogens known as natural selective estrogen receptor modulators.

The reduced size and lumen of the breast acini of rats treated with tamoxifen or tamoxifen + fulvestrant as compared to normal rats confirmed their antiestrogenic effects. Indeed, tamoxifen and fulvestrant are well known drugs that oppose the effects of estrogen in the mammary glands [16, 33]. The results obtained in the mammary glands with EECP at 150 mg/kg show that it has similar effects to fulvestrant at a lower magnitude. These results suggest that EECP did not alter the effect of tamoxifen on the mammary glands, which was similar to the results obtained in animals receiving tamoxifen only. The reproductive maturation that includes ovarian and follicle maturation is reached during puberty and is known as a prerequisite for fertility [34]. A significant (*p* < 0.001) accumulation of the number of primary and secondary follicles observed following the tamoxifen treatment as well as the significant (*p* < 0.01) decrease in the number of corpora lutea present in ovaries compared to normal rats suggests that tamoxifen inhibits the development of ovarian follicles. The EECP significantly decreased the number of primary follicles and increased the number of corpora lutea compared to ovaries of the tamoxifen-treated rats. This result suggests that EECP promotes the maturation of follicles, which in turn would promote ovulation and increase in fertility index as reported by Awounfack et al. [35]. In the same way, El-Sharkawy et al. [36] showed that propolis induces the development of ovarian follicles in rats exposed to an ovarian toxic. Ovarian cysts were observed in rats treated with tamoxifen only; this is in accordance with the work of Karim et al. [37], who reported hyperplasia of granulosa cells in the ovaries of rats treated with tamoxifen. EECP at the high dose (150 mg/kg) reduced these cysts burden.

No significant change was observed in the relative weight of organs which are indicators of the potential adverse effects of drugs. All animals grew normally during the 2 months of the experiment; however, the body weights of all animals treated with tamoxifen were significantly lower than those of normal rats. These results are in accordance with numerous studies and could be explained by the anorexigenic action of tamoxifen [20, 38].

## 5. Conclusion

This study showed that tamoxifen exhibited estrogenic effects on the uterus, which led to endometrial hyperplasia. EECP protected the rats against tamoxifen-induced endometrial hyperplasia, as evidenced by a significant decrease in the uterine wet weight; endometrial height; and expression of PCNA, Bcl-2, and VEGF proteins as well as a significant increase in the expression of Bax and caspase-3 proteins compared to the Tamox group. EECP did not change the effects of tamoxifen on the breast. In summary, the Cameroonian propolis which is efficacious in preventing breast cancer can also be a good complementary medicine to prevent tamoxifen-induced endometrial cancer in tamoxifen users.

## Figures and Tables

**Table 1:** Effects of 56-day oral treatment with EECP on some protein levels expressed in the endometrium of the ovaries of intact rats exposed to tamoxifen.

	NOR	Tamox	Ful + Tamox	EECP (mg/kg) + Tamox		
				16.5	50	150
Bcl-2	12.14 ± 1.3	16.56 ± 1.77^#^	14.25 ± 0.69	16.42 ± 0.87^#^	13.54 ± 0.74	12.88 ± 1.52^*∗*^
Bax	19.07 ± 1.9	14.73 ± 1.07^#^	21.60 ± 2.04^*∗∗∗*^	13.88 ± 1.29	15.57 ± 1.13	17.98 ± 1.16^*∗*^
VEGF	11.22 ± 0.9	14.54 ± 0.58^#^	15.10 ± 0.63	16.67 ± 0.78	11.34 ± 1.27^*∗*^	10.85 ± 1.07^*∗∗*^
Caspase-3	13.5 ± 0.72	14.52 ± 1.36	13.73 ± 1.78	14.01 ± 0.69	16.48 ± 0.91^*∗*^	18.42 ± 0.73^*∗∗*^
PCNA	7.12 ± 0.72	10.11 ± 0.94^#^	6.07 ± 1.06^*∗∗*^	10.62 ± 1.46^#^	7.75 ± 0.94	7.04 ± 0.55^*∗*^

NOR: rats treated with distilled water as normal control; Tamox: rats treated with tamoxifen only at the dose of 10 mg/kg BW as negative control; Ful: rats treated with the pure antiestrogen fulvestrant at the dose of 300 *μ*g/kg BW as positive control; EECP: rats treated with the ethanol-extracted propolis from Cameroon at doses of 16.5, 50, and 150 mg/kg BW as test groups. All rats apart from those of the normal group (NOR) were concomitantly administered tamoxifen at the dose of 10 mg/kg BW for 8 weeks to induce endometrial hyperplasia. Significance compared to NOR group: ^#^*p* < 0.05; significance compared to Tamox group: ^*∗*^*p* < 0.05, ^*∗∗*^*p* < 0.01, ^*∗∗∗*^*p* < 0.001.

**Table 2:** Effects of ethanol-extracted Cameroonian propolis on the ovaries of intact rats exposed to tamoxifen.

Items	NOR	Tamox	Ful + Tamox	EECP (mg/kg) + Tamox		
				16.5	50	150
*Types of follicles*						
Primary follicle	4 ± 1.63	16 ± 0^###^	3 ± 0.81^*∗∗∗*^	2 ± 0.63^*∗∗∗*^	0 ± 0^*∗∗∗*^	2 ± 0.63^*∗∗∗*^
Secondary follicle	0.93 ± 0.58	6 ± 1.14^###^	4.84 ± 0.65	4.88 ± 1.14	6.85 ± 1.55	5 ± 0.26
Tertiary follicle	3 ± 0.81	2.33 ± 0.68	4.5 ± 0.94	1.4 ± 0.4	1.62 ± 0.21	1.4 ± 0.4
De Graaf follicle	2 ± 0.36	1.6 ± 0.6	2.2 ± 0.58	2 ± 0	1.12 ± 0.11	2.8 ± 0.66
Corpora lutea	6.25 ± 1.47	1.75 ± 0.42^##^	4.74 ± 1.33^*∗∗*^	1 ± 0	2.25 ± 0.56	5.2 ± 1.01^*∗∗*^

*Cysts*						
Number of rats with cysts	0/6	2/6	0/6	2/6	2/6	1/6

NOR: rats treated with distilled water as normal control; Tamox: rats treated with tamoxifen only at the dose of 10 mg/kg BW as negative control; Ful: rats treated with the pure antiestrogen fulvestrant at the dose of 300 *μ*g/kg BW as positive control; EECP: rats treated with the ethanol-extracted propolis from Cameroon at doses of 16.5, 50, and 150 mg/kg BW as test groups. All rats apart from those of the normal group (NOR) were concomitantly administered tamoxifen at the dose of 10 mg/kg BW for 8 weeks to induce endometrial hyperplasia. Significance compared to NOR group: ^##^*p* < 0.01, ^###^*p* < 0.001; significance compared to Tamox group: ^*∗∗*^*p* < 0.01, ^*∗∗∗*^*p* < 0.001.

**Table 3:** Effects of ethanol-extracted Cameroonian propolis on some organ wet weights of rats exposed to tamoxifen.

Organs	NOR	Tamox	Ful + Tamox	Propolis (mg/kg) + Tamox		
				16.5	50	150
Liver	35154.35 ± 2443.31	31629.69 ± 1743.96	31775.94 ± 1356.68	34465.65 ± 1600.66	31308.05 ± 3110.49	29960.88 ± 1274.52
Lungs	8049.97 ± 218.69	8242.59 ± 297.05	8055.40 ± 420.15	8662.95 ± 676.63	6931.63 ± 398.52	6948.66 ± 353.29
Spleen	3640.16 ± 260.89	2845.29 ± 190.55	3460.98 ± 479.86	3535.42 ± 363.46	3496.95 ± 368.61	2929.93 ± 171.96
Adrenals	359.91 ± 42.09	373.98 ± 26.03	289.38 ± 9.38	365.07 ± 19.85	337.28 ± 28.48	316.70 ± 16.44
Kidneys	6232.45 ± 495.42	6769.13 ± 429.19	6666.26 ± 274.20	7555.93 ± 486.37	6626.18 ± 340.74	6609.92 ± 353.80
Femur	2784.48 ± 244.97	2974.92 ± 107.33	2404.77 ± 86.05	2725.13 ± 225.16	2557.32 ± 178.13	2731.94 ± 135.08
Brain	7549.21 ± 472.69	9140.60 ± 348.10^#^	8092.52 ± 354.54	8567.41 ± 510.52	8592.23 ± 427.23	8586.46 ± 309.92
Ovaries	526.11 ± 42.87	387.79 ± 22.73	381.40 ± 63.82	404.61 ± 48.35	468.00 ± 58.30	361.06 ± 53.91
Stomach	9437.52 ± 613.18	9609.25 ± 753.10	9453.80 ± 301.13	9300.52 ± 449.56	9251.86 ± 439.42	9322.35 ± 443.84
Heart	2965.42 ± 306.94	3572.65 ± 232.78	3810.40 ± 118.33	3869.70 ± 234.81	3762.17 ± 234.81	3508.87 ± 74.18

NOR: rats treated with distilled water as normal control; Tamox: rats treated with tamoxifen only at the dose of 10 mg/kg BW as negative control; Ful: rats treated with the pure antiestrogen fulvestrant at the dose of 300 *μ*g/kg BW as positive control; EECP: rats treated with the ethanol-extracted propolis from Cameroon at doses of 16.5, 50, and 150 mg/kg BW as test groups. All rats apart from those of the normal group (NOR) were concomitantly administered tamoxifen at the dose of 10 mg/kg BW for 8 weeks to induce endometrial hyperplasia. Significance compared to NOR group: ^#^*p* < 0.05.

## Data Availability

The data used to support the findings of this study are included within the article.

## Abbreviations

ANOVA: Analysis of variance
Bax: B cell lymphoma 2 associated X protein
Bcl-2: B cell lymphoma 2
c-Myc: Cellular-myelocytomatosis
DAB: Diaminobenzidine liquid tetrahydrochloride
ER: Estrogen receptor
HEK293T: Human embryonic kidneys cells
HER2: Human epidermal growth factor receptor 2
IHC: Immunohistochemical
PBS: Phosphate-buffered saline
PCNA: Proliferating cell nuclear antigen
PRKCD: Protein kinase C delta
Tamox: Tamoxifen
VEGF: Vascular epidermal growth factor
SEM: Standard error of the mean
SERM: Selective estrogen receptor modulator
SQSTM1: Sequestosome 1.
