# Correction to “Ethanol‐Extracted Cameroonian Propolis Counteracts Tamoxifen‐Induced Endometrial Hyperplasia by Modulating Apoptosis and Proliferation‐Regulating Proteins in Intact Wistar Rats”

**DOI:** 10.1155/ecam/9829670

**Published:** 2025-12-26

C. F. Awounfack, S. Zingué, B. Koumabas, et al., “Ethanol‐Extracted Cameroonian Propolis Counteracts Tamoxifen‐Induced Endometrial Hyperplasia by Modulating Apoptosis and Proliferation‐Regulating Proteins in Intact Wistar Rats,” *Evidence-Based Complementary and Alternative Medicine* (2022): 2684742, https://doi.org/10.1155/2022/2684742.

In the article, there is an error in the title, as the proteins were evaluated in the uterus, not ovaries. In light of this,

“Ethanol‐Extracted Cameroonian Propolis Counteracts Tamoxifen‐Induced Endometrial Hyperplasia by Modulating Apoptosis and Proliferation‐Regulating Proteins in the Ovaries of Intact Wistar Rats”

Has been corrected in the article to

“Ethanol‐Extracted Cameroonian Propolis Counteracts Tamoxifen‐Induced Endometrial Hyperplasia by Modulating Apoptosis and Proliferation‐Regulating Proteins in Intact Wistar Rats”

We apologize for this error.

